# Generating a Bessel-Gaussian beam for the application in optical engineering

**DOI:** 10.1038/srep18665

**Published:** 2015-12-22

**Authors:** Xiuxiang Chu, Quan Sun, Jing Wang, Pin Lü, Wenke Xie, Xiaojun Xu

**Affiliations:** 1 School of sciences, Zhejiang Agriculture and Forestry University, Lin’an 311300, China; 2 College of Optoelectronic Science and Engineering, National University of Defence Technology, Changsha 410073, China; 3 Science and technology on integrated information system laboratory, Institute of software Chinese Academy of Sciences, Beijing, 100190, China; 4 Beam control key lab of Chinese academy of Sciences, the Institute of optics and electronics the Chinese Academy of Sciences, Chengdu, 610209, China

## Abstract

Bessel beam is the important member of the family of non-diffracting beams and has many novel properties which can be used in many areas. However, the source of Bessel beam generated by the existing methods can be used only in a short distance due to its low power. In this paper, based on the coherent combining technology, we have proposed a method which can be used to generate a high-power Bessel beam. Even more, we give an innovative idea to form vortex phase by using discontinuous piston phase. To confirm the validity of this method, the intensity evolution of the combined beam and the Bessel-Gaussian beam at different propagation distance have been studied and compared. Meanwhile, the experimental realization has been discussed from the existing experimental result related to the coherent combining technology.

In recent years, many new interesting properties of non-diffracting beam were intensively investigated. The particular attention was focused on the applications in both the fundamental and applied physics^1^,^2^,^3^,^4^. For example, the self-healing property of Airy beams leads to a new feature in optical micromanipulation^5^, a holographically shaped and scanned Bessel beam not only reduces scattering artefacts^6^, but also simultaneously increases the quality of the image and penetration in dense media^7^,^8^. The interesting properties and successful application of non-diffracting beam in micro-imaging have attracted many attentions in other fields. In free-space, quantum communication or between quantum processing systems, Bessel beam offers advantages to overcome the losses of quantum entanglement between photon pairs^9^,^10^. In the applications in atmosphere, such as in optical communication, non-diffracting beam exhibits more resilience against perturbations^11^,^12^,^13^.

Since Bessel beam is the important member of the family of non-diffracting beams, many methods have been proposed to generate this beam^14^,^15^,^16^,^17^,^18^,^19^,^20^,^21^,^22^,^23^. However, because of beam spreading and the loss of energy during propagation, the energy of Bessel beam generated by those methods for long distance propagation is still a problem, such as in quantum and optical communication. In optical engineering, phase locking and coherent combining technology can be used to generate laser source of high brightness by several laser sources^25^,^26^,^27^,^28^,^29^,^30^,^31^,^32^. In the past few years, coherent beam combining have multiplied their output power reaching kilowatts^24^,^33^. In this paper, we investigate the validity for generating Bessel-Gaussian (BG) beam by coherent combining technology. Meanwhile, we suggest an idea to form a vortex phase by using discontinuous piston phase which can be used in the generation of many other vortex beams^23^.

## Results

A Bessel beam which is the exact solution of the Helmholtz equation has infinite energy and cannot be generated in practice. Research shows that BG beam with finite energy has some properties as that of Bessel beam^9^,^10^. In this paper, the expression for the BG beam at the initial plane (z = 0) is given as


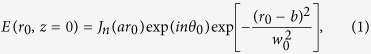


where *J*_*n*_ represents the *n*th-order Bessel function of the first kind, (*r*_0_, *θ*_0_) is a pair of polar coordinates, *a* is a scale factor, *w*_0_ is the waist width of Gaussian part. Because a high order Bessel-beam (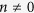) is a hollow beam with many rings, a Gaussian annular aperture is introduced to restrict the beam where *b* is the radius of the annular.

**Figure 1:**
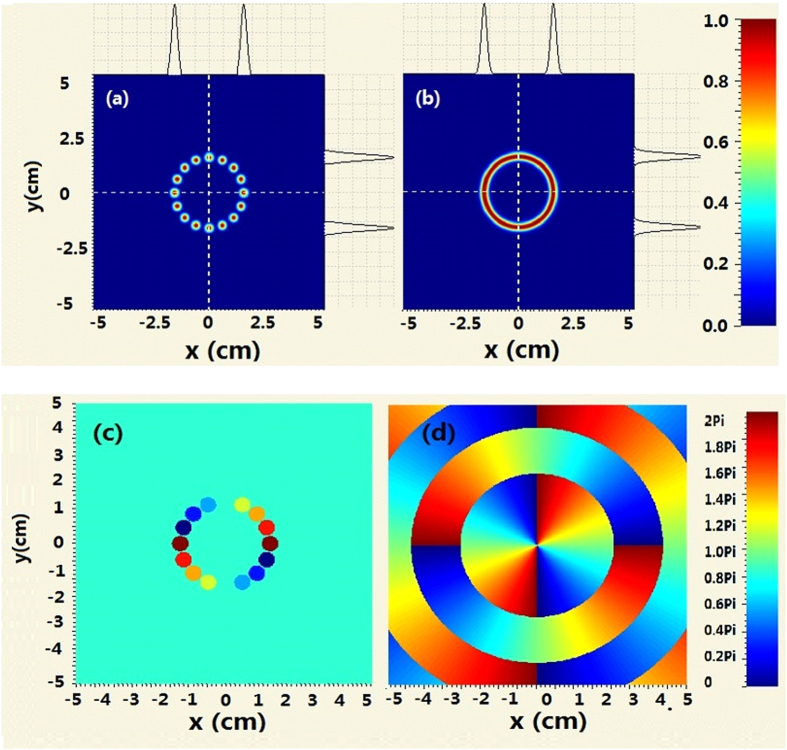
Comparison of the intensity distribution and phase map between coherently combined beams and second-order BG beams (**a**) intensity distribution of coherently combined beams (**b**) intensity distribution of second-order BG beams (**c**)phase map of coherently combined beams (**d**) phase map of second-order BG beams.

At first, we compose a second-order BG beam (n = 2) by using 16-Gaussian beams. The intensity distribution and phase map of the combined beams and the second-order BG beam is given in Fig. 1 where a = 200m^−1^, w_0 _= 0.0025 m and b = 0.015 m. To compose the second-order BG beam, as shown in Fig. 1a, the 16-Gaussian beams are given as


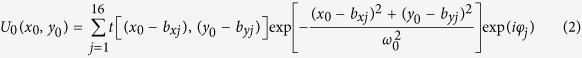


where 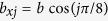, 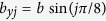 is the central position of the *j*th-Gaussian beam, ω_0 _= 0.0025 *m* is the waist width of each Gaussian beams, 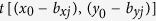 is the hard aperture and is expressed by


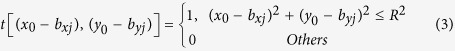


Here *R*_ _= 1.1 ω_0_ is the radius of the hard aperture. The vortex phase is composed approximately of piston phases, namely 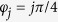, see Fig. 1c. In practice, the different piston phase of each Gaussian beam can be modulated by the phase locking technology.

**Figure 2:**
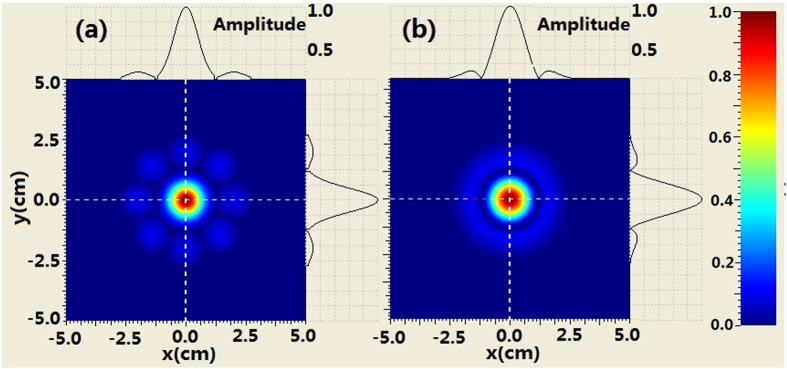
Comparison of the amplitude distribution between coherently combined beams and zero-order BG beams, (**a**) coherently combined beams (**b**) zeroth-order BG beams.

Secondly, we can also generate the zeroth-order BG beam by the coherently combined beams. The amplitude distribution of the zeroth-order BG beam and combined Gaussian beams are shown in Fig. 2 where the BG beam is also expressed in Eq.(1) with *n*_ _= 0, *a*_ _= 200 m^−1^, *w*_0_ = 0.015 m and *b*_ _= 0. The combined beams are composed of 9 Gaussian beams and are expressed as


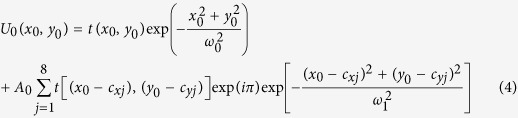


where 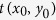 is the hard aperture with radius 0.012 *m* located at the center, 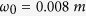, 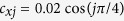
*m*, 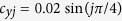
*m* is the central position of the *j*th-Gaussian beam, ω_1 _= 0.006 m is the waist width of the Gaussian beam located at the position of the first ring, 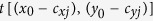 is the hard aperture with radius 0.007 *m* located at (*c*_*xj*_, *c*_*yj*_), *A*_0 _= 0.1 and 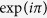 express the maximum amplitude and the phase of the 8-Gaussian beams located at the location of the first ring, respectively.

## Discussion

**Figure 3:**
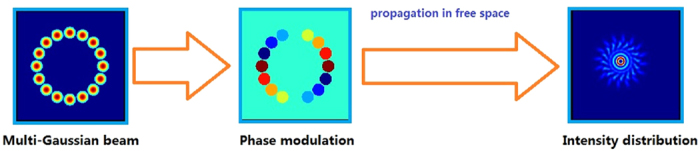
Simulation block diagram for the generation and propagation of combined second-order BG beam.

To confirm the validity of the method, the comparison of the intensity profiles between the combined beam and the BG beam at different propagation distance is simulated by the *see-light software* developed by *National University of Defense Technology* and *Institute of software Chinese academy of Sciences* where the wavelength is 550 nm. The *see-light software* is based on the angular spectrum approach and two-dimensional fast Fourier transform. Simulation block diagram for the generation and propagation of combined second-order BG beam is shown in Fig. 3. Namely, 16-Gaussian beams are arranged as in Fig. 1a. The phase of each Gaussian beam is modulated by different piston phase where 
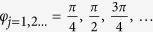
.

**Figure 4:**
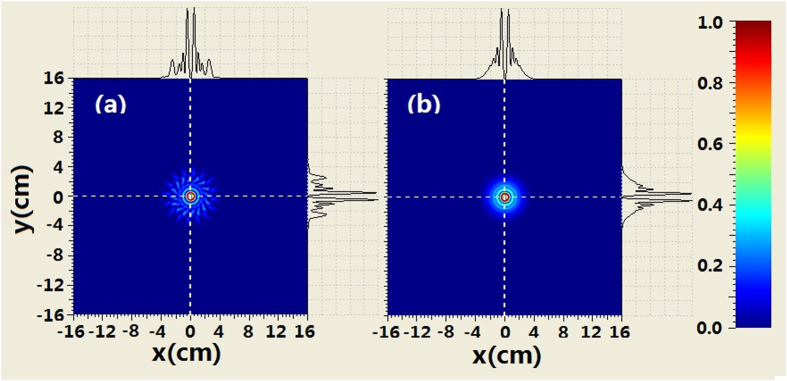
Comparison of the intensity evolution between coherently combined beams and second-order BG beams where z = 250 m, (**a**) coherently combined beams (**b**) second-order BG beams.

**Figure 5:**
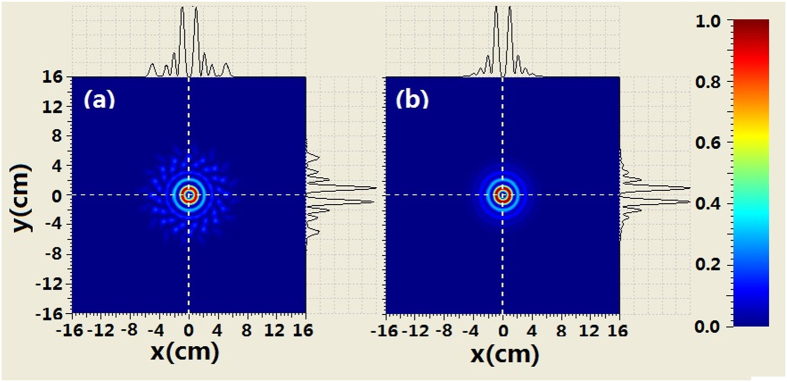
Comparison of the intensity between coherently combined beams and second-order BG beams where z = 500 m, (**a**) coherently combined beams (**b**) second-order BG beams.

**Figure 6:**
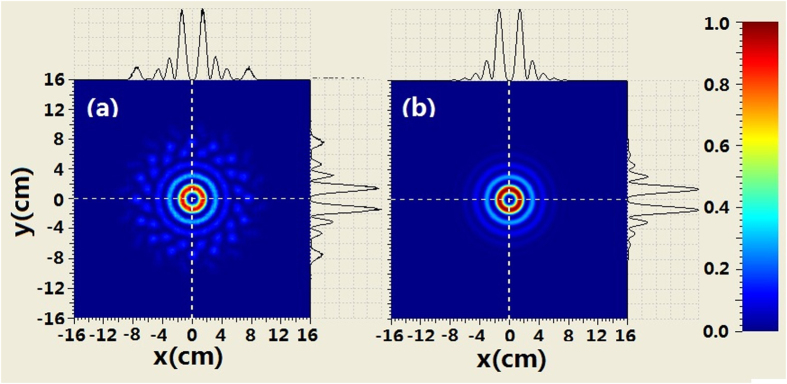
Comparison of the intensity between coherently combined beams and second-order BG beams where z = 750 m, (**a**) coherently combined beams (**b**) second-order BG beams.

The comparison of the intensity profiles between combined BG beam and second-order BG beam is shown in Fig. 4 when the propagation distance is 250 m. It can be seen from Fig. 4 that the evolution of the intensity at the center area for the combined beam is similar to that of the BG beam. For example, the second-order ring near the main ring appears. However, there are many petals outside the rings of combined beam which is different from the BG beam. When increasing propagation distance, such as *z*_ _= 500 *m* we can see from Fig. 5 that the coherently combined beam has the same multi-ring shape as that of the BG beam. However, the petals outside the rings of the combined beam still exist. Further increasing the propagation distance (z_ _= 750 m), the intensity profiles of combined beam is very similar to those of the BG beam at the central area. Meanwhile, petals outside the rings of the combined beam still remain, as shown in Fig. 6.

**Figure 7:**
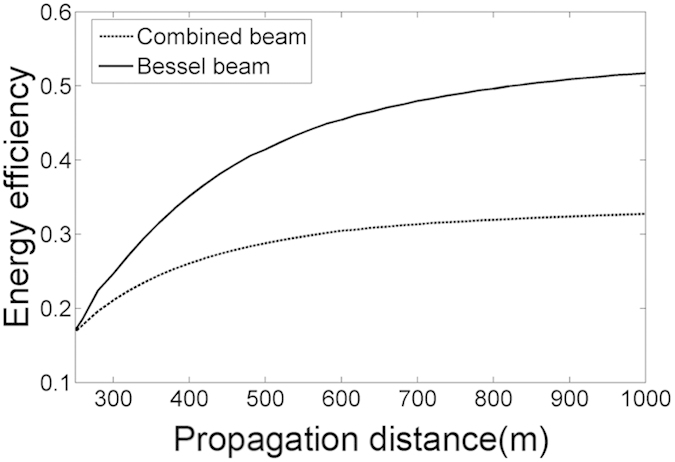
Variation of the energy efficiency of the combined beam during propagation.

The evolution of the intensity for coherently combined beams shows that the petals outside the rings always exist. Comparing with the BG beams, the petals will cause the loss of the energy. The variation of the loss of the energy with propagation distance is shown in Fig. 7. It should be pointed out that the energy efficiency of the combined beams is defined as the ratio between the energy of the first ring and the total energy. Because our purpose is to generate BG beam, the energy efficiency between the BG beams and the combined beams is compared.

Because the multi-ring-shaped beam has formed when the propagation distance is larger than 200 m, the energy efficiency after 200 m has been plotted. From Fig. 7 we can see that the energy efficiency is very lower when the propagation distance is shorter. With the increase of propagation distance, the energy efficiency gradually tends to a constant value. However, the energy efficiency of the combined beam is much less than that of the BG beam.

**Figure 8:**
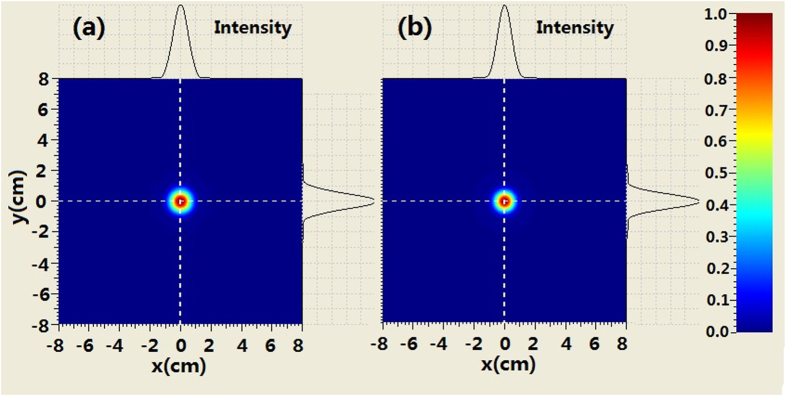
Comparison of the intensity evolution between coherently combined beams and zero-order BG beams where z = 250 m, (**a**) coherently combined beams (**b**) zero-order BG beams.

**Figure 9:**
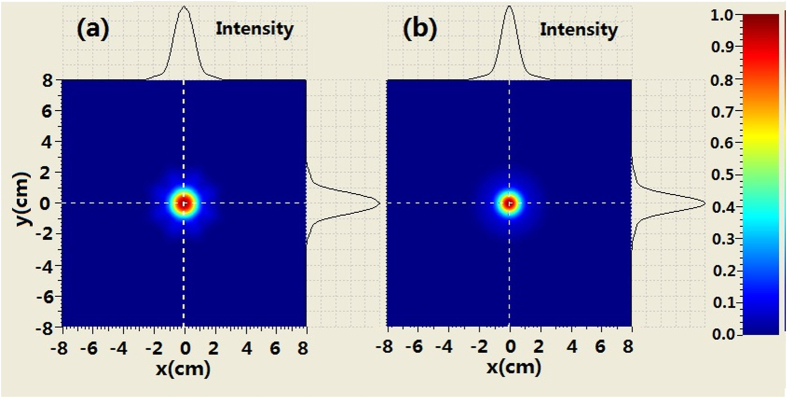
Comparison of the intensity evolution between coherently combined beams and zero-order BG beams where z = 500 m, (**a**) coherently combined beams (**b**) zero-order BG beams.

**Figure 10:**
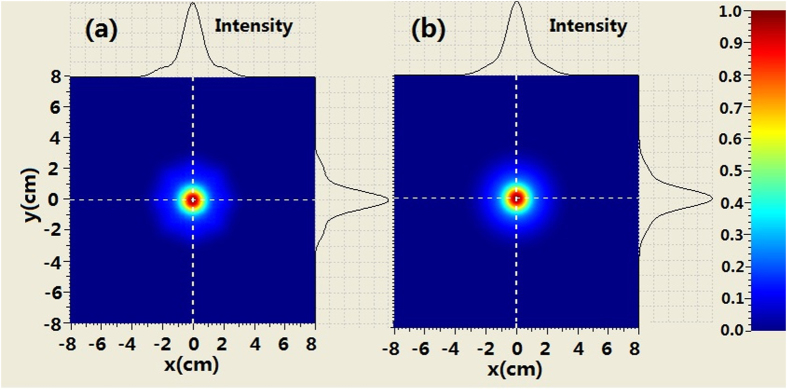
Comparison of the intensity evolution between coherently combined beams and zero-order BG beams where z = 750 m, (**a**) coherently combined beams (**b**) zero-order BG beams.

The evolution of the combined zeroth-order BG beam and the corresponding BG beam also are simulated by the *see-light software* where the wavelength is 550 *nm*. The intensity distributions with different propagation distance are shown in Figs 8, 9, 10. It can be seen that the main spots of the combined zeroth-order BG beam is very similar to that of the BG beam during propagation. For example, with the increase of propagation distance, the central spots of the two types of the beams spread with the same speed, and the second ring come together with the central spots. Meanwhile, the intensity uniformity of the combined beam becomes better during propagation.

**Figure 11:**
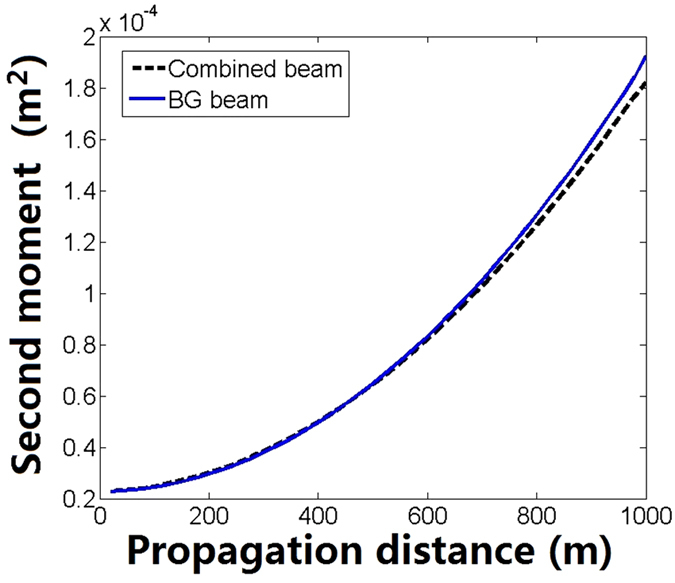
Comparison of the second moment of the intensity between coherently combined beams and zero-order BG beams.

To compare qualitatively the evolution of the intensity for the two beams, the variation of the second moment of intensity is shown in Fig. 11 where the second moment of intensity is defined as


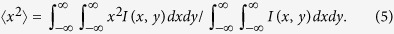


Here 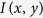 is the intensity distribution. We can see that the variational trend of the second moment for the combined beam is the same as that of the BG beam. However, the difference is growing with the increase of propagation distance.

## Methods

**Figure 12:**
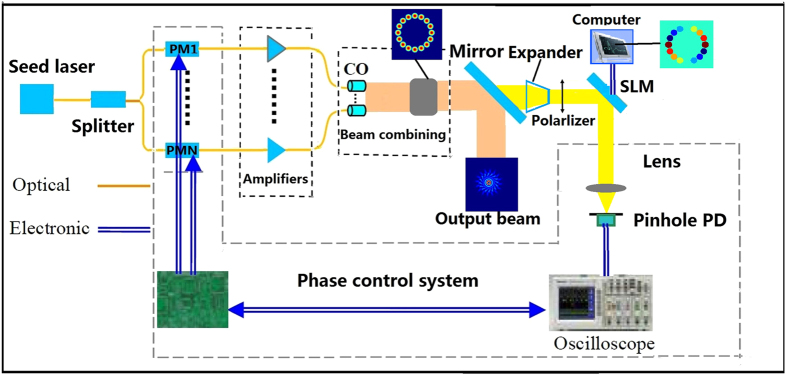
Experimental setup for the generation of a BG beam by the coherent combining technology where PM is phase modulators array and CO is collimators array.

The simulation software is developed by *National University of Defense Technology, China* and *Institute of software Chinese academy of Sciences*. The principle of the software is based on the angular spectrum theory and fast Fourier transform. Because fiber lasers have lots of advantages over other kinds of lasers, such as high conversion efficiency, convenient heat management and excellent beam quality^32^,^33^, to confirm the validity of the technique proposed in this paper, an experimental setup for the generation of 2-order BG beams by fiber lasers is depicted in Fig. 12.

A seed laser is split into 16 channels by the splitter. Each beam which phase is controlled by the phase modulators (PM) is amplified by multiple cascaded amplifiers. Then these beams pass through the collimators (CO) array and the beam combiner to reform the arrays as shown in Fig. 1a. The combined arrays are split into two beams by the mirror with high reflectivity. Most of the laser energy is reflected by the mirror. The residual transmitted laser which phase is modulated by the SLM is focused by a lens and coupled into the photoelectric sensor with a pinhole to offer the feedback signal to the phase control system. The optical phase in each channel is decoupled by the phase control system and the control signal is added to the phase modulator, so that the optical phase in each channel is the same when the phase control system is in a closed loop^32^,^33^. Because the phase of the residual transmitted laser is modulated by the discontinuous piston phase as shown in Fig. 1c, the reflected laser has the conjugate phase of SLM.

Existing results show that the fiber pigtailed phase modulator and acoustics-optics frequency shifter make possible the active phase control with MHz-level control bandwidth which could be used for phase locking in the experiment^32^,^33^. Many experiments show that this method can obtain a satisfied result in beam combining with high precise phase control. Since not only the distribution of the intensity given in Fig. 1a, but also the discontinuous piston phase as shown in Fig. 1a can be generated, these results confirm the validity of the method.

## Additional Information

**How to cite this article**: Chu, X. *et al.* Generating a Bessel-Gaussian beam for the application in optical engineering. *Sci. Rep.* **5**, 18665; doi: 10.1038/srep18665 (2015).
